# Insomnia: Especially in Relation to Dyspepsia
*The first article appeared on August 30.


**Published:** 1913-09-13

**Authors:** Guthrie Rankin

**Affiliations:** Physician to the *Dreadnought* and Royal Waterloo Hospitals.


### INSOMNIA: ESPECIALLY IN RELATION TO DYSPEPSIA.*

By GUTHRIE RANKIN, M.D., F.R.C.P., Physician to the *Dreadnought* and Royal Waterloo Hospitals.

Many devices have been suggested as means of procuring sleep for those who, apparently healthy and free from strain of any kind, are yet unable to command it. We are all familiar with many of the popular nostrums, some of the more well known of which have been recounted by Wordsworth, himself a victim of insomnia, in the following lines:—

A flock of sheep that leisurely pass by
One after one; the sound of rain, and trees
Murmuring; the fall of rivers, winds, and seas,
Smooth fields, white sheets of water, and pure sky;
I've thought of all by turns, and still I lie
Sleepless; and soon the small birds' melodies
Must hear, first uttered from my orchard trees,
And the first cuckoo's melancholy cry.
Even thus last night, and two nights more I lay
And could not win thee Sleep! by any stealth:
So do not let me wear to-night away:
Without thee what is all the morning's wealth?
Come, blessed barrier between day and day,
Dear mother of fresh thoughts and joyous health!

As a rule, but one to which there are many exceptions, those who wish to sleep soundly should cease hard work for an hour or more before going to bed. Other conditions being favourable, it is said that if you think of nothing you will sleep, but it is difficult for most men completely to control thought by an effort of the will. The drowsy effect of monotony is a well-known sedative; the enumeration of figures, the falling of rain, the lap of the sea on a shingly beach, the droning voice of an uninteresting and prosy reader are examples familiar to us all. On this principle it has been suggested that sleep may be induced by an arrangement in the bedroom whereby drops of water are allowed to fall at regular intervals into a resonant vessel, but this and all other such ingenious contrivances are, for the most part, useful just for so long as they are novel, and apply to the individual only. Similarly it has been urged that the system of tiring the eyes by directing them to a small beam of light placed in front and slightly above their level is an inducement to sleep. This, again, is a recommendation of individual application, and borders on the larger question of hypnotism, which, however useful, cannot be discussed now.

In mild cases of insomnia change of scene, a sea voyage, moderate exercise in the open air, temporary withdrawal from the ordinary activities of life are, each and all of them, minor measures not to be neglected. It must also be remembered that in those who sleep lightly, trivial discomforts may be enough to produce sleepless nights. Cold feet, an unwise evening meal, an absorbing game, a heated discussion, a postponed hour of retirement, and a hundred other small departures from the accustomed routine may be responsible for a bad night. It is quite remarkable how this is exemplified in the common experience of a sleepless night on Sunday, when the usual activities of the week are interrupted by comparative freedom from care, more than the usual amount of repose, and frequently also more than the average allowance of food and drink.

Massage, especially when practised thoroughly and rapidly over the abdomen, a hot bath, a mustard foot-bath, a wet-pack applied to the trunk of the body, a hot drink fortified by alcohol are all homely measures which, by provoking a temporary anæmia of the brain, are conducive to the acquisition of refreshing sleep. Many forms of electricity have also been requisitioned with varying degrees of success.

When all these minor methods fail, recourse to drugs of the narcotic and hypnotic class becomes a necessity. The most certain of all sleep-producers is opium or morphia, and in most cases where pain is the cause of the insomnia its advantages outweigh those of any other drug though its use in chronic sleeplessness is not to be advocated because of the fear that the opium habit may be engendered. Nevertheless, even in these cases, one or two initial hypodermic injections of morphia, given by the doctor or nurse and not left to the patients' own discretion of administration, often provides for a better result from whatever subsequent form of hypnotic is chosen. For such a purpose the dose should be a quarter or a third of a grain, and the good effect will be intensified by combination with one-hundredth of a grain of atropine.

When sleep is prevented by muscular or neuralgic pains, aspirin, antipyrine, antifebrin, exalgin, phenacetin, and a long list of similar analgesics may, by relieving the pains, assist in the induction of sleep. They often act most efficaciously when combined with a small quantity of narcotic. Here are two illustrative prescriptions which are frequently successful under such circumstances: (*a*) Aspirin and phenacetin, of each ten grains; Dover's powder, four grains; (*b*) antipyrine and salicin, of each ten grains; heroin one-tenth of a grain.

Of all the hypnotics the bromides are the least harmful, and a full dose of one or other of the salts is sometimes sufficient to procure calm and refreshing sleep. Thirty or forty grains should be taken at bedtime, and repeated during the night if the patient awakes. The bromide is in many cases more reliable if given with hop and sumbul in this combination: Bromide of sodium, thirty grains; tincture of hop and tincture of sumbul, of each half a drachm; chloroform water to one ounce. Many compounds of bromide have been advocated instead of the simple salt, and there are two which are specially worthy of mention—namely, bromural, which may be given in a cachet of ten grains or more; and dilute hydrobromic acid, in doses of from half to one drachm.

Next to the bromides, chloral hydrate is one of our most valuable hypnotics. It has always seemed

* The first article appeared on August 30.

to me that the depressing influence of chloral has been made too much of, and that we are often debarred from its administration by a wholly unjustifiable fear of the possible harm to the circulation which may ensue upon its use. If it is chosen only for cases where there is no debility of the cardiac walls, and given in judicious dosage, it is reliable and excellent. It may, in many instances, be advantageously combined with one of the bromides: chloral hydrate, twenty grains; potassium bromide, forty grains; tincture of henbane, thirty minims; and peppermint water to one ounce constitutes a hypnotic mixture of the greatest service when selected for a suitable patient. Though the combination of morphia with chloral and bromide is, as a rule undesirable, it is none the less true that if the above mixture fails to secure a good night, the addition to it of twenty minims of the liquor morphiæ hydrochloratis frequently secures the desired result. After a few evenings the morphia may be withdrawn, when the original mixture will be found to be of itself sufficient. Of the many chloral compounds which are on the market the following are useful: Bromidia, which is a mixture of chloral, bromide, cannabis indica, and hyoscyamus, may be given in doses of from one to two drachms; chloretone, obtained by heating acetone and chloroform with caustic potash, may be given in doses of ten to twenty grains; chloralamide, a combination of chloral with formamide, in doses of thirty to sixty grains; and chloralose, a mixture of chloral and glucose, in doses of from four to ten grains.

Hyoscine is one of our most powerful hypnotics and is said never to induce a habit, but owing to its toxicity care must be observed in its administration. It is of special advantage in cases where there is great restlessness but no valvular disease, and is best administered hypodermically in doses of from one two-hundredth to one one-hundredth of a grain of the hydrobromide.

Cannabis indica, though sometimes used as a hypnotic, is uncertain in its action; it is distinctly sedative in its effect upon the nervous system, but, except in combination with other drugs of the hypnotic type, cannot be relied upon as conducive to a better habit of sleep.

Of other hypnotics, reference can only be made to those that are better known because they have been tried and proved. Sulphonal is the type of a pure hypnotic. It possesses no analgesic properties, but in insomnia, uncomplicated by pain, it still remains one of our best and most trustworthy remedies. It ought to be given in a full dose of thirty grains, and is suitable, in proportionate quantity, for children. It is tasteless and may be taken in hot soup or milk, or, better still, in a strong glassful of hot brandy or whisky and water. Trional is an allied drug which, in twenty to thirty grain doses for adults and in proportionate quantities for children, is a reliable substitute for sulphonal. It is said to be less cumulative and more rapid in its effect, and certainly cases are met with where it succeeds brilliantly in securing sound sleep when sulphonal has proved quite useless. Veronal has recently come much into vogue, and is used by the public with a happy ignorance of its power for evil. It is given in doses of from seven to ten grains, but it is less reliable than either sulphonal or trional, and is much more liable to produce unpleasant and even dangerous consequences. If it were not for its evil taste and the unpleasant odour which it communicates to the patient's breath, paraldehyde is one of the safest and most effective of all the simple hypnotics. In doses of from one to two drachms it may be given with full confidence that, whatever the state of the heart and circulation, it will produce no depressing effect even if it should fail to secure the desired amount of sleep. Its disagreeable taste is best concealed by combination with tincture of orange or oil of cinnamon. When one after another of the simple hypnotics given alone fail, it will be found that they may sometimes be combined so as to achieve a satisfactory result. The explanation which has been offered of this well-known fact is that when two or more medicaments find separate receptors in the cells of the body the physiological effect of each drug in an ordinary dose will be obtained, and is of greater value than a double dose of either drug given alone. The following examples are quoted in illustration: (*a*) Trional, twenty grains; apomorphia, one-twentieth of a grain; (*b*) sulphonal, twenty grains; bromural, ten grains. (*c*) Paraldehyde, one drachm; bromide of potassium, thirty grains. (*d*) Chloralamide, thirty grains; morphia, one-eighth of a grain. (*e*) Bromide of ammonium, thirty grains; tincture of valerian, one drachm; spirits of chloroform, twenty minims. (*f*) Veronal, five grains; phenacetin, ten grains; codeia, half a grain.

Of the vast army of recent hypnotics no mention need be made here. Few of them are better than those that are now accepted on their proved merits; some are dangerous except in selected cases, and many are quite ineffective. We cannot glance at the advertising columns of the medical, and even the lay, papers without being struck by the versatility of the chemist and the simple faith of the public. The demand for them obviously exists, else the supply would not be forthcoming in such luxuriant abundance, and the inference would seem to be either that humanity is more sleepless than it used to be, or that it is less tolerant of occasional disturbance of its night's rest.

The widespread addiction to hypnotics without any reference to the actual need or to the form of drugs most suited to personal requirements is nowadays a popular practice which is greatly to be deplored. It is not only unscientific but unwise, and is one of the many influences which are, in the present stage of our civilisation, weakening the adaptability of the human nervous system. The sooner it is recognised that drugs of the hypnotic and narcotic class should be only procurable on the prescription of a qualified medical practitioner the better it will be not only for the individual but for the nation.

